# Financial Impact of Deep Sternal Wound Infections After Coronary Surgery: A Microcosting Analysis

**DOI:** 10.21470/1678-9741-2022-0261

**Published:** 2023-07-18

**Authors:** Bianca Maria Maglia Orlandi, Omar Asdrúbal Vilca Mejia, Evelinda Marramon Trindade, Fabio B Jatene

**Affiliations:** 1 Department of Cardiovascular Surgery, Instituto do Coração (InCor), Hospital das Clínicas da Faculdade de Medicina da Universidade de São Paulo (HCFMUSP), São Paulo, São Paulo, Brazil; 2 Cardiovascular Surgery Service, Hospital Paulistano, São Paulo, São Paulo, Brazil; 3 Rede Paulista de Avaliação de Tecnologias de Saúde, Coordenação de Ciência, Tecnologia e Inovação da Secretaria de Estado da Saúde de São Paulo, São Paulo, São Paulo, Brazil

**Keywords:** Coronary Artery Bypass, Propensity Score, Patient Discharge, Mediastinitis, Electronics

## Abstract

**Introduction:**

Deep sternal wound infections (DSWI) are so serious and costly that hospital services continue to strive to control and prevent these outcomes. Microcosting is the more accurate approach in economic healthcare evaluation, but there are no studies in this field applying this method to compare DSWI after isolated coronary artery bypass grafting (CABG). This study aims to evaluate the incremental risk-adjusted costs of DSWI on isolated CABG.

**Methods:**

This is a retrospective, single-center observational cohort study with a propensity score matching for infected and non-infected patients to compare incremental risk-adjusted costs between groups. Data to homogeneity sample was obtained from a multicentric database, REPLICCAR II, and additional sources of information about costs were achieved with the electronic hospital system (Si3). Inflation variation and dollar quotation in the study period were corrected using the General Market Price Index. Groups were compared using analysis of variance, and multiple linear regression was performed to evaluate the cost drivers related to the event.

**Results:**

As expected, infections were costly; deep infection increased the costs by 152% and mediastinitis by 188%. Groups differed among hospital stay, exams, medications, and multidisciplinary labor, and hospital stay costs were the most critical cost driver.

**Conclusion:**

In summary, our results demonstrate the incremental costs of a detailed microcosting evaluation of infections on CABG patients in São Paulo, Brazil. Hospital stay was an important cost driver identified, demonstrating the importance of evaluating patients’ characteristics and managing risks for a faster, safer, and more effective discharge.

## INTRODUCTION

**Table:** 

Abbreviations, Acronyms & Symbols				
BMI	= Body mass index		ICD-10	= International Classification of Diseases, Tenth Revision
CABG	= Coronary artery bypass grafting		ICU	= Intensive care unit
CCS	= Canadian Cardiovascular Society		LOS	= Length of stay
CI	= Confidence interval		NCSP	= NOMESCO Classification of Surgical Procedures
COPD	= Chronic obstructive pulmonary disease		NYHA	= New York Heart Association
CSQI	= Cardiac Surgery Quality Initiatives		PSM	= Propensity score matching
DRG	= Diagnosis-related groups		REPLICCAR	= Registro Paulista de Cirurgia Cardiovascular
DSWI	= Deep sternal wound infections		SD	= Standard deviation
FAPESP	= Fundação de Amparo à Pesquisa do Estado de São Paulo		STS ACSD	= Society of Thoracic Surgeons Adult Cardiac Surgery Database
HbA1C	= Hemoglobin A1C			

Deep sternal wound infections (DSWI) as a result of open-heart surgery are so serious and costly that hospital services continue to strive to control and prevent these outcomes^[1,2]^. The prevalence of DSWI in coronary artery bypass grafting (CABG) patients varies between 1% and 4% worldwide^[3,4]^. And multiple risk factors are associated with infections in cardiac surgery, such as female sex, age, diabetes, obesity, renal failure, smoking, steroid use, and chronic obstructive pulmonary disease (COPD)^[1,4,5]^.

In Brazil, a study reported the total cost of CABG per patient of US$7,992.55^[6]^. Prior estimates of the cost of hospitalizations after surgical infections vary widely across hospitals, states, and regions, and range from US$24,000 to US$58,000^[7-9]^.

Quality improvement practices were first implemented by Ernest Codman, who migrated from the technology industry to clinical practice and collaborated to improve outcomes, even with high-complexity procedures involved^[10]^. Throughout the study of the Registro Paulista de Cirurgia Cardiovascular (REPLICCAR), our team participates in the Cardiac Surgery Quality Program to identify opportunities for quality improvement in cases in which high-cost and resource-intensive frequently preventable outcomes might occur. Quality interventions do not necessarily imply increased hospital costs, as it focuses on the optimization of an existing organizational process. Some examples are medical care focuses on patients, protocols based on the best available evidence, decisions made by a multidisciplinary team, real-time data to show quality improvement protocols, benefits of interventions and their impact on patients, and education leading to positive changes^[10,11]^.

There is a need to establish appropriate priorities between patients’ groups with an effective selection for treatment within particular characteristics, based on the risk of complications and chance of survival, rehabilitation, and acceptable quality of life. Risk scores have become an important tool in patient assessment, including factors such as age, the severity of heart disease, and co-morbidity in the type of cardiac procedure. However, most scoring systems are used to predict mortality, and further refinement to specific morbidity risk scores is necessary to predict both outcome and hospital costs^[11]^.

Microcosting is the more accurate method to describe economic evaluation in healthcare. It can provide the most precise approach of deriving interventional costs because it involves direct enumeration and costing of each interventional input, such as nurse or pharmacist time for the procedure, and capital inputs, such as facilities space. The process includes three stages: (1) identification of all resources involved in the provision of care (*e.g.*, human resources, consumables/materials); (2) accurate measurement of each resource (*e.g.*, time and motion studies); (3) valuation of the resources used^[12]^. Only a few studies reported this method in cardiac surgery^[11,13-16]^, none of them used the microcosting approach to estimate hospital costs for DSWI as a severe and costly complication in postoperative patients of cardiac surgery.

In that view, DSWI and mediastinitis represent a preventable outcome with a resource-intensive environment. The purpose of our study was to estimate the cost of DSWI and mediastinitis in a sample of isolated CABG patients from a referenced cardiac hospital in São Paulo, Brazil.

## METHODS

This is a retrospective observational cohort study using a single center for the microcosting analysis of patients with DSWI and without complications after isolated CABG as the first cardiac procedure. Data were obtained from the REPLICCAR II database, which was a multicentric cohort study performed by voluntary participant hospitals between August 2017 and June 2019. The variables included in REPLICCAR II were defined using the Society of Thoracic Surgeons Adult Cardiac Surgery Database (STS ACSD) collection tool (version 2.9 - 2017). Approximately 760 variables were collected preoperatively, intraoperatively, and postoperatively, and included risk factors, clinical and laboratory characteristics, and complications of surgery. The data were collected using a secure web application for building and managing online surveys and databases, the REDCap platform (Research Electronic Data Capture, https://www.project-redcap.org/).

The Comissão de Ética para Análise de Projetos de Pesquisa (ethical committee board) approved the study under the protocol number CAPPesq: 2.507.078 and received funding from Fundação de Amparo à Pesquisa do Estado de São Paulo (FAPESP) (CNPq PPSUS FAPESP 2016/15163-0); patient consent was not required.

### Criteria and Definitions

DSWI were classified according to the Centers for Disease and Control National Healthcare Safety Network (or CDC NHSN) criteria and definitions^[17]^. Profound infections (involving fascia, muscle layers, and/or deep soft tissue) and organs/spaces infections (mediastinitis/osteomyelitis) were included. Superficial infections were excluded due to discrepancies in treatment compared to deep infections.

During the REPLICCAR II study, the surgical surveillance system followed patients until 30 days after surgery, where they received a call after discharge asking about their recovery. Thus, patients that persisted in the hospital and developed infections were included in this analysis. The groups were divided into control, deep infections, and, separately, mediastinitis/osteomyelitis.

### Adjusted Costs

To conduct our analysis, the values were corrected by the inflation costs variation on the General Market Price Index of the Fundação Getúlio Vargas adopting the study period for calculation (August 2017 to July 2019). Also, the dollar quotation variation on the period was considered to estimate the costs in American dollars. The data is available online for public consultation (http://ipeadata.gov.br/Default.aspx).

### Missing Data

The REPLICCAR II database includes a total of nine eligible centers in São Paulo (Brazil), with data of patients that underwent CABG from 2017 to 2019. The missing data in the database was mostly related to clinical characteristics, missing completely at random, and it was < 30%. Multiple imputations by chained equations (or MICE) were performed in R Studio software with 10 imputations (n=4085 observations, *P*=161 variables). After imputation, data from a single center was captured to guarantee a homogenous sample comparison between infected and non-infected patients using propensity score matching (PSM).

### Propensity Score Matching

PSM considered matching nearest neighbor. The predictors included patients with infections who died until 30 days of follow-up. Dependent variables were preoperative in-hospital duration, gender, body mass index, prior family history of coronary disease, previous myocardial infarction, hypertension, peripheral artery disease, renal failure, dyslipidemia, insulin dependence, previous percutaneous intervention, COPD, surgical status, angina Canadian Cardiovascular Society classification, and intraoperative blood transfusion. The treatment group included patients with wound interventions after surgery.

### Microcosting Analysis

To include the costs of each component and apply a microcosting evaluation, we took additional data from the hospital system (Si3) to get detailed information related to all resources available, such as additional hospital stays, utilities and medications, re-interventions, clinical, laboratory and image tests, bandage, etc.

Statistical analyses were conducted using STATA 16.1 (StataCorp, College Station, Texas, United States of America) software package. Costs and length of stay (LOS) were described with average and 95% confidence intervals (CI). Linear regression was performed to evaluate factors related to increased costs between groups. Analysis of variance was used to compare differences between groups, and *P*<0.05 was considered significant.

## RESULTS

A total of 1,120 CABGs were performed in the hospital between 2017 and 2019. The DSWI prevalence during the period was 4.7% (n=53), and prevalence of mediastinitis/osteomyelitis was 1.4% (n=16). The STS risk score for mortality was on average 1.14% (standard deviation [SD]=0.9) in the DSWI group and 1.07% (SD=0.8) for patients without infections; and the STS model for DSWI (including mediastinitis) was 0.19% in average (SD=0.07) on the infected and 0.12% (SD=0.8) on non-infected patients. Seven patients died within 30 days (10.7%) - one in the same hospitalization (in-hospital mortality) and another six were re-admitted until 30 days after CABG (five with mediastinitis and one with deep infection). After PSM, 66 patients were allocated between groups. Sample characteristics after matching groups are described in Table 1.

**Table 1:** Sample characteristics after propensity score matching between control and infected patients (DSWI and mediastinitis) after CABG (Instituto do Coração, Hospital das Clínicas, Faculdade de Medicina, Universidade de São Paulo, 2020).

Characteristics	Control (n=30) n (%)	DSWI (n=26) n (%)	Mediastinitis (n=10) n (%)	*P*-value
Age, average ± SD	62.1±10.2	62.4±9.8	66.8±5.9	0.379
Female sex	14 (40)	16 (45.7)	5 (14.3)	0.527
Diabetes	20 (44.4)	19 (42.2)	6 (13.3)	0.728
Insulin-dependent	2 (20)	5 (50)	3 (30)	0.136
Hypertension	24 (43.6)	22 (40)	9 (16.4)	0.823
Dyslipidemia	19 (48.7)	13 (33.3)	7 (18.0)	0.521
BMI > 30 (Kg/cm^2^)	10 (45.5)	9 (40.9)	3 (13.6)	1.000
Chronic obstructive pulmonary disease	1 (50)	0 (0)	1 (50)	0.282
NYHA ≥ III	5 (33.3)	5 (33.3)	5 (33.3)	0.080
Angina CCS 4	6 (50)	5 (41.7)	1 (8.3)	0.833
Peripheral artery disease	4 (50)	4 (50)	0 (0)	0.607
Previous myocardial infarction	9 (33.3)	14 (51.9)	4 (14.8)	0.212
Elective status	15 (50.0)	11 (42.3)	6 (60.0)	0.620
Preoperative ICU admission	14 (40)	16 (45.7)	5 (14.3)	-
Days in hospital before surgery	5.6	5.8	4	0.698
Preoperative exams				
HbA1c (%), average ± SD	6.4±1.1	7.6±2.1	7.4±1.3	0.024
Glucose (mg/dL), average ± SD	136±46	166±80	143±63	0.226
Creatinine (mg/dL), average ± SD	1.0±0.2	1.2±1.1	1.8±1.6	0.071

Chi-square or Fisher’s exact test or analysis of variance BMI=body mass index; CABG=coronary artery bypass grafting; CCS=Canadian Cardiovascular Society; DSWI=deep sternal wound infections; HbA1C=hemoglobin A1C; ICU=intensive care unit; NYHA=New York Heart Association; SD=standard deviation

**Fig. 1:**
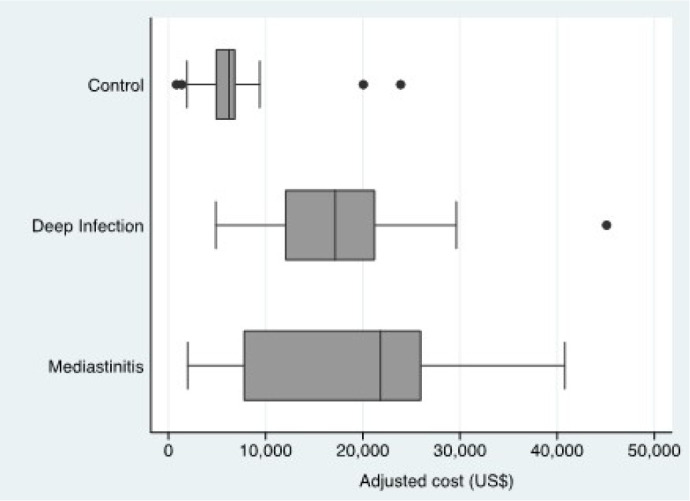
Financial impact on coronary artery bypass grafting total costs related to deep infections and mediastinitis (Instituto do Coração, Hospital das Clínicas, Faculdade de Medicina, Universidade de São Paulo, 2020).

Figure 1 describes the hospitalization total costs between groups. As expected, deep infection increased the costs by 152%, and mediastinitis increased it by 188%. The mean cost for the control group was $6,863 (SD=4,615); for deep infections, it was $17,329 (SD=8,471); and for mediastinitis it was $19,805 (SD=13,383).

**Fig. 2:**
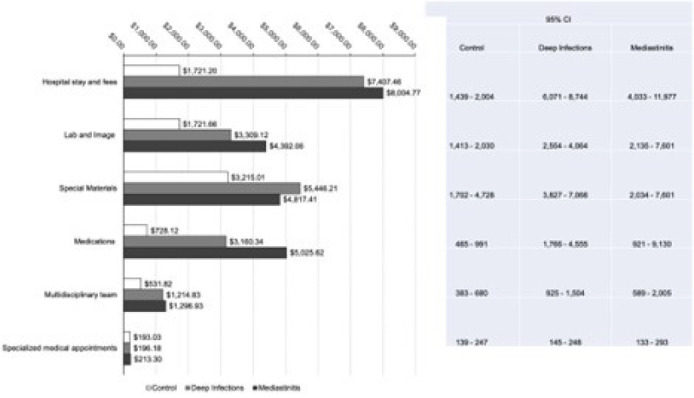
Average cost of deep sternal wound infections after coronary artery bypass grafting divided by departments using microcosting approach. Bars indicate the average cost (95% confidence intervals [CI] are shown on the right) (Instituto do Coração, Hospital das Clínicas, Faculdade de Medicina, Universidade de São Paulo, 2020).

Figure 2 describes the microcosting analysis with the average financial impact of infections on costs for each hospital department involved with the cardiac surgery process. Hospital stays, special materials, laboratory and image exams, medications and nutrition, and multidisciplinary labor were the costliest services for infected patients and were statistically different compared to the control group. However, the cost driver identified by the multiple linear regression was the hospital stay (b=0.0001; adj R2=0.48; 95% CI .00006-.0001; *P*=0.000).

Considering time in intensive care unit (ICU), 19.2% and 40% of patients with deep infections and mediastinitis were readmitted for intensive care. The mean LOS in ICU after readmission was 13 days in the deep infections group and 21 days in the mediastinitis group. The groups were statistically different in total postoperative duration, where the control group total LOS was 9.5 days, the DSWI was 37 days, and the mediastinitis was 59 days (*P*=0.000).

## DISCUSSION

Little information is available on the final impact of infections after major cardiac surgery. DSWI results in significant patient morbidity and consumes considerable resources and hospital LOS^[18,19]^. The main purpose of this study was to provide an accurate description of how much infections (DSWI and mediastinitis) increase costs related to open surgical treatment in CABG patients. It should come as no surprise that there is a strong positive correlation between LOS and hospital costs. What is of interest is the relative importance of each complication to the cost structure of isolated CABG patients and the hospital’s ability to create policymakers to predict and manage these circumstances^[9]^.

Still, previous studies confirm our findings and reinforce the need for preventive methods^[18]^. Graf et al. report that the main proportion of costs related to DSWI concerns to ward care, additional interventions, and prolonged ICU care^[20]^. Besides the prolonged hospital stay, mortality and morbidity rates during treatment are high. Gib et al. report that using preconditioning wounds, such as vacuum therapy, could reduce mortality by 22% compared to time-only procedures^[21]^.

On the other hand, Brandt et al.^[22]^ published a structured literature review with data from 14 countries, including Brazil. The paper explores the burden of surgical infections after CABG. However, infection rates varied considerably between settings, with infections occurring in 2.8% (the United Kingdom) to 10.4% (the Netherlands) of CABG procedures, while the costs per surgical infection varied between $8,172 (Brazil) to $54,180 (Japan). Important limitations of this analysis include uncertainty about the surveillance methods, criteria and definitions, and superficial infections.

Economic evaluation studies in surgery frequently use “top-down” or “gross-costing” approaches and, usually, are based on healthcare resource groups, which can be used to estimate the average cost per inpatient episode for groups of surgical procedures^[11]^. The diagnosis-related groups (DRG) were a framework created for monitoring hospital activity and efficiency and thus to control the increasing hospital costs better. The unique DRG to which any procedure is assigned is based on disease, comorbidities, and complications, as recorded by the International Classification of Diseases, Tenth Revision (ICD-10). Treatment codes are given by the NOMESCO Classification of Surgical Procedures (NCSP); the national DRG code is automatically calculated by a computer algorithm introducing the ICD-10 codes and NCSP codes. The algorithm is constructed to allocate the most complicated patients to the lowest DRG code number^[6]^. However, there are several limitations related to these methods, such as the need to compare two different surgical procedures within the same group or evaluate a modification/actualization to an existing process^[12]^.

Brazilian economic and health systems databases are challenging to manage costs or quality because the ICD-10 is biased and does not represent clinical characteristics. Data collection is very scarce, and most observations are summaries per region, so individual observations aren’t possible using the National Database. In this study, we opted to use the REPLICCAR II database due to all limitations in Brazilian sources.

Prediction models designed for specific outcomes consistent with real population parameters may provide more accurate information about patients and hospital resources. Developers of performance measures will also be expected to promote their measures to health systems and payers. Another tough work would be to best educate consumers about why the measures are important to increase quality. Fortunately, such measure-promotion efforts will be synergistic with registry-promotion activities. Through advocacy for wider measure adoption, models can simultaneously promote the use of their performance measures and the registries that report those measures, thereby furthering the goals of patient-centered care^[22]^.

The STS models, for example, are widely used by Cardiac Surgery Quality Initiatives (CSQI) programs, such as the Virginia CSQI and the American Association for Cardiac Surgery^[23]^. The Virginia CSQI evaluates adherence to clinical and process metrics derived from performance measures from the STS ACSD. This voluntary consortium of 17 hospitals and 13 cardiac surgical practices in Virginia (United States of America) identified quality improvement opportunities. It tracked patient outcomes but also found options for cost containment, such as improved patient outcomes and decreased resource utilization^[24,25,26]^.

In 2018, the Centers for Medicare & Medicaid Services announced that the bundled payments for care improvement advanced. The bundled payments, also described as episode payment models, are designed to move toward value-based care by incentivizing providers to go above the target price for an episode, including those that arise from complications and hospital readmissions. The idea is to support quality programs that invest in practice innovation and care redesign to better coordinate and reduce expenditures while improving the quality of care^[27,28]^.

In addition, Brescia et al.^[27]^ (2020) reported that assessing tradeoffs between spending and quality is essential for success in bundled reimbursement models. Although the authors didn’t evaluate the tradeoffs, they made a retrospective observation of 33 nonfederal hospitals in Michigan (United States of America) and identified determinants of variability between hospitals.

Implementing quality programs may represent the key to success for continuous improvement results.

### Limitations

Several limitations can be related to this observational, retrospective design, which cannot account for all potential confounding variables in this situation. The study criteria included only sternal infections, and the follow-up and readmissions data were considered. However, in our scenario, this study provides an estimated cost for infections in isolated CABG. It allows us to use clinical data in healthcare management to provide excellent quality based on knowledge. We must note that we based our data on a single Brazilian institution, and costs may not be generalized for other facilities and country regions.

## CONCLUSION

In summary, our results demonstrate the incremental costs of a detailed microcosting evaluation of infections on CABG patients in São Paulo, Brazil. Hospital stay was an important cost driver identified, demonstrating the importance of evaluating patients’ characteristics and managing risks for a faster, safer, and more effective discharge.

## Data Availability

The data generated during the current study are not publicly available due to ethical restrictions; patients did not consent to their deidentified data being publicly shared, but these data are available on reasonable request to the Scientific Committee Director Renata do Val (renata.doval@incor.usp.br; https://www.incor.usp.br/sites/incor2013/index.php/16-pesquisa/comissao-cientifica/158-fale-conosco).
